# A Conceptual Model of Help-Seeking by Black Americans After Violent Injury: Implications for Reducing Inequities in Access to Care

**DOI:** 10.1007/s11121-022-01429-6

**Published:** 2022-08-30

**Authors:** Caterina G. Roman

**Affiliations:** Department of Criminal Justice, Temple University, 1115 Polett Walk, 5th Fl Gladfelter Hall, Philadelphia, PA USA

**Keywords:** Barriers to service, Crime victimization, Help seeking, Police interaction, Racial inequities, Police reporting, Violent injury

## Abstract

Many inequities exist in serving and supporting Black survivors of violent crime. A key question in reducing inequities in care after victimization is whether police first responders and other formal system providers identify the victim as an “offender” and/or someone who is “undeserving” of supports. These labels and associated biases can directly reduce access to supports through a variety of mechanisms that include police withholding information about one’s rights as a victim, among other direct and indirect barriers to social and health services. Unaddressed financial, mental, and physical health consequences of victimization contribute to poorer health outcomes later in life. This paper seeks to bring together the extant research on help-seeking, discrimination in criminal legal system functioning, and barriers to victim services by synthesizing these discrete threads into a theoretically and empirically informed conceptual model that captures the range of factors that shape Black Americans’ decision to report their victimization to the police and subsequent help-seeking. Qualitative and quantitative data from a purposive sample of 91 Black victims of community violence is used to ground the developing model. The conceptual model can help lay the foundation for research that seeks to remedy the marked mismatch between the prevalence of violent victimization and help-seeking among Black Americans. Research findings can be applied to guide policies and programming to reduce inequities in care for victims of violence.

## Introduction

Violent crime victimization is a significant problem in the USA, causing a range of physical and psychological harms to individuals and costing billions annually (Miller et al., 1993) in victim costs, criminal legal system costs, and intangible costs, such as pain and suffering, decreased quality of life, and psychological distress. Research shows that victimization increases the likelihood of substance use and misuse and suicidal behavior (Hanson et al., 2010; Zimmerman & Posick, 2016). Furthermore, individuals injured in violent crime incidents are at an elevated risk of re-victimization (McCart et al., 2010).

Black Americans experience these harms disproportionately compared to white Americans. The 2020 National Crime Victimization Survey (NCVS) found that serious violence is 42% higher against Black Americans than white Americans (Morgan & Thompson, 2021). Although the overall rates of violent victimization in the USA have been decreasing in recent years, the rate of violent victimization among Black persons has not changed significantly. Furthermore, studies indicate that Black Americans are exposed to more psycho-social and environmental stressors than other racial groups (Green et al., 2019). These stressors include, but are not limited to, systemic racism and police over-surveillance (Bell, 2017; Smith, 2003; Treadwell et al., 2010). Young Black men hold a unique status here, not simply because they are more likely to be violently victimized than individuals from other demographics (NIJ, 2014), but also because Black men’s high levels of victimization intersect with the multitude of short- and long-term collateral consequences of mass incarceration, racial discrimination, low socioeconomic status (SES), and low-quality healthcare (Hughey, 2015) to place them in the demographic with the highest risk for poor health outcomes and the largest reductions in life expectancy (Jones-Eversley et al., 2020; Treadwell et al., 2010).

With regard to healthcare and social support services post-victimization, studies indicate that there is high unmet need among Black citizens for basic victim services, such as victim advocacy, victim compensation, or referral to counseling. Data from the National Crime Victimization Survey reveal that the lowest rates of receipt of services from victim service agencies are among individuals who are Black (compared to whites), younger (ages 18–34 compared to those older than 34), male (compared to female), those living in urban areas (compared to rural), and those who do not report assaults to the police (Langton, 2011). Add the government-funded service exclusions for some victims due to criminal legal system involvement and exclusions for non-cooperation with the police, and service access and receipt are likely even lower. Essentially, states have a range of policies that exclude victims from state-funded financial compensation and related services because of past and current participation in criminal activity and non-cooperation with police for their crime investigations. A huge amount of discretion lies with the reporting police officers and case detectives. These exclusions reduce the likelihood Black survivors of violence would receive medical and mental health services, criminal legal system advocacy and support, and government-funded compensation related to their victimization. This wide gap in services likely compounds health disparities and contributes to the high mortality rate for Black Americans and particularly Black men (Noonan et al., 2016).

A key question in reducing inequities in care after violent victimization is whether first responders and other criminal justice and healthcare system providers identify a Black victim as “undeserving” and if this label influences subsequent victim treatment, including limiting access to services and medical care. In under-resourced urban areas where violence tends to be higher and police surveillance is frequent, racism and explicit and implicit biases are likely to influence post-injury interactions between Black survivors of violence and institutional actors and compound barriers to care (Patton et al., 2019; Rich, 2009; Ullman, 2010). Furthermore, cultural orientations about law and justice among Black Americans may reflect structural marginalization and legal estrangement (Bell, 2017, 2020) that greatly reduce the likelihood that those harmed will turn to the police for help or go to the hospital for care (Brayne, 2014). The fact that access to victim services is frequently predicated on victim cooperation with police and rely on law enforcement’s determination of cooperation further complicates the relationship between service access and the “deserving” victim (Hipple et al., 2019). These front-door barriers to care have been linked to reductions in victim services support and later healthcare (Newmark, 2006; Roman et al., 2022). But the paths taken by Black individuals after violent injury are not well understood. Awareness of and access to victim services in the direct aftermath of victimization have rarely been studied within the larger context of help-seeking, let alone with an eye toward understanding how historical and contemporary racism and the related societal and neighborhood-level influences associated with racism (e.g., mass incarceration) play a role. There is limited research on help-seeking pathways after victimization in general, and by extension, the health disparities that might result due to differential access or engagement in care, likely because the different disciplines with an interest in reducing violent victimization—criminology, sociology, public health, social work, prevention science, psychology, nursing, and psychiatry—all have their own methodologies and research agendas.

This article seeks to bring together the extant research on help-seeking, social determinants of health, discrimination in criminal legal system functioning, and victim services and synthesize into a theoretically and empirically informed conceptual model to facilitate a deeper understanding of the contextual factors that shape Black victim help-seeking after violent injury. A draft conceptual model of Black victim help-seeking is first derived from reviewing the extant literature across disciplines, and then the draft model is re-assessed against empirical data capturing the perceptions of 91 Black individuals injured in incidents of community violence. Although help-seeking is a multi-faceted construct and definitions often vary depending on the specific health-related topic of focus, for the purposes of this article, help-seeking is defined as the behavior or dynamics associated with how people access and obtain formal services in the immediate aftermath of their crime victimization. Here, formal help-seeking includes the decision to report the victimization to the police. This encompassing definition including police reporting is purposeful; it enables integration of the disparate literatures, including the impacts of systemic discrimination within the criminal legal system on Black Americans, in effort to stimulate research that can help inform and evaluate best practices for supporting people of color injured by community violence (Xie & Baumer, 2019). Inherent in the model is the assertion that Black populations use different help-seeking pathways largely because of societal, cultural, and neighborhood factors entwined with their racial identities. Prevention science-oriented conceptual models provide a framework to advance systematic study of a wide and encompassing array of risk and protective factors associated with well-being. Systematic study will yield evidence-informed research implications that engender policies and programs designed to address the previously unaddressed financial and physical and behavioral health consequences of victimization that contribute to poor health outcomes. Essentially, a prevention-based focus on Black survivors of violence that addresses multiple levels of context (e.g., societal, neighborhood), which is rare across public safety efforts in the USA, could minimize the short and long-term harms from injury and trauma, help improve levels of trust across residents and criminal legal system actors, and ultimately impact the cycle of violence.

## Help-Seeking in the Context of Victim Services and Post-injury Healthcare

A full-fledged, formal system of supports for victims of crime did not fully develop until the mid-1980s, as the victim rights movement grew. Formal support relevant to victim services can be provided by actors within the criminal legal system (e.g., police officers, district attorneys), social service agency staff, medical services personnel, crisis hotline workers, mental health professionals, clergy and faith-based organizations, and victims of crime advocates. It is these supports that are often available and funded through the Crime Victims Fund (CVF), a fund that is authorized through the federal Victims of Crime Act (VOCA). The federal Office of Victims of Crime (OVC) manages the VOCA funds, which awards state victim compensation programs. The state programs can make direct payment to victims or funds go to state VOCA administrators to award to direct service providers (Newmark, 2006). The program can cover loss of earnings, out-of-pocket expenses for medical care, counseling and home healthcare services, loss of earnings, and crime scene clean-up.

As the formal support system for victims of crime grew, the literature on victim help-seeking began to emerge, not surprisingly showing that the overwhelming majority of victims of violent crime were not seeking support through *formal* victim services (McCart et al., 2010) and that those who are Black, on average, are less likely to seek formal services than non-Black victims (Alvidrez et al., 2008; Langton, 2011; McCart et al., 2010; Monterrosa, 2021; Sered, 2019). Furthermore, to date, the overwhelming majority of studies on the help-seeking behavior of victims has focused on female survivors of interpersonal violence (IPV) (NIJ, 2014), resulting in a wide gap in our knowledge of help-seeking for victims of community violence (i.e., violence typically committed in public areas by individuals who are not intimately related to the victim). Community violence has different dynamics than those related to domestic or dating violence; the dynamics that identify and portray the “victim” and “offender” are quite different as well (NIJ, 2014).

Labeling a victim as an “offender,” even without any explicit intent to do so, may shape the path to victim services, as some studies show that victims of domestic violence and sexual assault endure the label and biases and resultant barriers to care, associated with the “undeserving” female. Given the long arm of mass incarceration and its collateral consequences and the growing studies highlighting the impacts of systemic racism throughout the criminal legal system, Black men and women are particularly vulnerable to biases and prejudices from law enforcement first responders (Brunson & Miller, 2006; Sered, 2019; Tahouni et al., 2015). Myths and truths related to what has been deemed by some scholars as the “victim-offender overlap” may greatly affect access to social and health services. Police officers are most often the first to the scene of a victimization; officers are trained to respond to the injured person’s needs and provide information that explains their rights as victims and a list of resources where they can seek further help (Newmark, 2006).

Although racism and discrimination toward Black survivors of violence by law enforcement may not be widely documented in the research literature, studies show that Black men and women encounter racism and discrimination in hospitals and across ambulatory health services regardless of victim status (i.e., victim/non-victim). One systematic review of studies on implicit racial and ethnic biases in healthcare concluded that all but one study found implicit biases did exist and that these biases negatively affected healthcare outcomes (Hall et al., 2015). Survey research on patient perceptions of discrimination suggests that minority patients perceive higher levels of racial discrimination than non-minorities (LaVeist et al., 2000; Lillie-Blanton et al., 2000) and uninsured Black respondents are more likely to report racial bias in healthcare compared with their privately insured counterparts (Stepanikova et al., 2008). Notably, it does not matter whether the discrimination is real or perceived; perceptions of discrimination shape behaviors regarding health and social service systems (Hall et al., 2015). These findings of widely encountered biases against Black individuals by healthcare professionals have serious implications for Black men injured by violence. Physician John Rich, in his book *Wrong Place, Wrong Time* (2009, 77), documents that trauma surgeons in the hospital where he worked often grouped injured young Black men into the category of “hardened drug dealers” simply judging by how the patient engaged with/spoke to the surgeon. His book highlights how these men are disenfranchised from the services needed, often have a distrust of hospitals, and hence, tend to avoid formal settings. Other research, including Patton and colleagues' study (2019) of Black survivors of gun violence who entered a Level 1 trauma center after their injury, confirms these findings and suggests that the interplay of social issues in urban areas (e.g., high poverty, high crime, high victimization rates, high levels of Black male criminal legal system involvement) may bring about augmented levels of racism and discrimination from healthcare providers and associated staff that, in turn, adversely affect short- and long-term outcomes for patients (Gallen et al., 2022).

## Ullman’s (2010) Ecological Model of Help-Seeking

Across the literature on victims of crime, researchers have often drawn from Ullman’s (2010) Integrated Ecological Model of Help-Seeking after Sexual Assault to help elucidate the array of contextual factors that influence help-seeking. Ullman’s (2010) model, developed specifically with regard to violent victimization, adapted Liang et al.’s (2005) feminist social ecological model of disclosure and help-seeking after IPV to be used in understanding help-seeking processes after rape/sexual assault. Liang and colleagues’ model, which has some foundation in Bronfenbrenner’s social ecological model (1979), brought needed attention to understanding that how the survivor interprets the situation, not simply what they do after the incident, has a strong influence on what follows. Their model made advances that allowed for gaining insight into the multi-level, non-recursive help-seeking factors and processes that ultimately impact health. Ullman’s model expanded Liang and colleagues’ work in separating out contextual factors from individual factors and clarified three post-victimization phases related to help-seeking: (1) problem definition; (2) disclosure and help-seeking; and (3) longer-term outcomes related to disclosure and help-seeking, such as health outcomes. The careful delineation of contextual (i.e., social climate, myths, norms, culture, gender inequality, and access and availability of services) in Ullman’s model provides a base to develop the model discussed herein of help-seeking for Black individuals injured by community violence. The extant literature reviewed above, and specifically the large differences between sexual assault and community violence, the documented differences in help-seeking behavior between men and women, and a growing literature on police–citizen interactions, police legitimacy, and the gaps in victim services with findings of differences across racial lines (Xie & Baumer, 2019), suggests the necessity of adapting Ullman’s model with the addition or expansion of a range of themes and factors of influence. Given page constraints, this article focuses only on the factors that precede the three phases of post-victimization help-seeking.

## Method

### Data Collection and Participants

The data for this study are derived from a larger, multiple-interview, mixed methods project on victims of community violence collected in Philadelphia, Pennsylvania, over the period from January 2018 to March 2019 and designed to understand why and how victims receive services and both formal and informal support after victimization. The data used for the current study include the first paired survey and semi-structured interview with victims of community violence. Participants included men and women between the ages 18 and 40 who experienced a violent injury from community violence within 12 months before study recruitment. Community violence is defined as predatory crimes that tend to occur outside, such as aggravated assault. The definition excludes violence perpetrated as part of a romantic or dating relationship. Respondents from all regions of Philadelphia were recruited by a team of five researchers representing diverse backgrounds and two formerly incarcerated street outreach workers. For interested participants, eligibility was determined immediately, and an interview was scheduled. The research team sought to conduct most interviews and surveys at the time of recruitment. All participants provided written consent. The survey/interviews were conducted in the offices of a violence reduction program or in quiet public settings. Participants were compensated $50 for each survey/interview. Recruitment yielded 103 eligible participants, of whom 94 (91.3%) considered themselves to be Black or part Black. Two interviews were not recorded, and one victimization was due to an assault by police; these respondents are not included in this analysis, resulting in a sample of 91 Black respondents.

The demographics of the sample, derived from the survey data, are as follows: three quarters (74.7%) are male; the average age is 26.8. Roughly a quarter (24.2%) had either been shot or stabbed with the remainder having serious injuries such as broken jaws and other broken bones, severe concussions, and internal injuries. Only 15.4% sought and received some type of victim service. Fewer than 5% received services from a victim advocate in the month following their incident, although roughly 10% received some type of help with the legal process. Only 4 individuals (4.4%) applied for victim compensation (note that these services categories are not mutually exclusive—in that the respondents utilizing a victim advocate, for instance, could be the same respondents accessing help with the legal process). With regard to victim compensation, roughly three quarters of respondents indicated that they did not know they could be financially compensated, although a small percentage of those who did not know about compensation also said they would not have applied for compensation even if they knew about it. Two individuals said they thought they did not qualify for compensation, so they did not apply. None of the respondents indicated they tried to apply and were denied. The overwhelming majority did not receive any type of counseling or treatment either in the 30 days following the incident or after those first 30 days.

### Procedure

The survey and interview protocols were developed after a deep review of the literature on help-seeking; coping after trauma; experiences of Black and other minority survivors of crime; access to victim services; and race, procedural justice, criminal justice contact/criminal justice involvement, and police interactions with victims. Similarities and differences between the literature and Ullman’s help-seeking model were assessed. The model on which coding was based (hereafter referred to as the draft conceptual model) also drew heavily from theoretical and empirical studies on interpersonal racial discrimination and health disparities and the intersection of race and criminal justice decision-making. In particular, we drew from the phenomenological variant of ecological systems theory (PVEST) (Spencer et al., 1997) and Monica Bell’s tripartite theory of legal estrangement (2017, 2020) because some of the factors elucidated in these theories were not part of Ullman’s model of help-seeking. PVEST incorporates concepts and processes that take into account that Black Americans must navigate dual roles as being Black and American, and hence, any behavioral outcomes framework should incorporate how individuals positively or negatively reorganize their self-perceptions in response to stereotypes and biases. Bell’s work, which has criticized social science for not understanding and appropriately conceptualizing the realities African Americans face on a daily basis due to their symbolic and structural marginalization, advances constructs that reflect the phenomenological ideas of PVEST and specifically describes three aspects of socio-legal processes (“legal estrangement”) that greatly influence how Black American perceive and interact with the criminal legal system: procedural injustice, vicarious marginalization, and structural exclusion. PVEST and Bell’s tripartite legal estrangement framework add important constructs at the societal level and the individual level.

In addition to the literature review, two focus groups and 8 semi-structured interviews were conducted with Black individuals injured by violence and victim services leaders to inform a draft conceptual model and the research protocols. Having a criminal record, general lack of awareness of victim compensation and related services, limited police follow-up post-victimization, antagonistic hospital, and police interactions were the key themes that arose from the focus groups and interviews. As a result of these research tasks, the semi-structured interview protocol used in the field focused on questions about reporting to the police, any police interaction, whether any agencies followed up or contacted the victim after the incident, general knowledge of available victim services, and the victim’s perceptions of any law enforcement response and how those perceptions may have influenced help-seeking. Probing was done to elicit detailed responses. The survey, which was conducted first, included questions on demographics, the injury sustained, personal social networks, the social and health services accessed (or not), and any perceived barriers. On average, the survey and interview together took between 45 min and 1.5 h to complete. A few in-depth interviews were as short as 12 min if the survivor did not have contact with the police or did not pursue services.

### Data Coding and Analyses

All recorded interviews were transcribed, and the quality was checked by reviewing every fifth interview recording and making necessary corrections. Transcripts were then de-identified and uploaded into ATLAS.ti version 8. Analyses combined directed, deductive, and inductive content analyses (Hsieh & Shannon, 2005). The deductive process centered on initially developing an a priori codebook in line with the draft conceptual model. After a deep review of the literature, to capture factors that precede the three phases of help-seeking, codes were grouped into three overarching model categories: (1) contextual level, social structure (i.e., criminal justice-related such as mass incarceration, economics, resources, and policies/laws); (2) contextual level, culture (i.e., racism, neighborhood context, and gender norms); and (3) individual level. A series of main (parent) and sub (child) codes were developed that were largely descriptive in nature as opposed to value-laden (Saldana, 2013). Descriptive codes were selected because this field of research is largely exploratory, and the research team felt imbuing value before a review of all codes together would be less thorough than allowing themes to arise as a part of the analytical process. This initial coding scheme was piloted and revised iteratively among the principal investigator (PI), a qualitative research assistant, and two research assistants who had conducted the interviews. Three four-page segments for each of three transcripts were selected for piloting by this larger group of researchers. Each segment was independently coded by each researcher, and after completion of a segment, the group would discuss their coding decisions and revise the codebook by adding or modifying codes and code definitions. Memos were used to denote any themes that did not fit under the original codes and subcodes. After three iterative rounds with all four coders and two additional tests to ensure these codes were exhaustive by the PI and the main qualitative researcher, a total of three additional main codes and 12 additional subcodes were added to the codebook. Although the research team had built the initial codebook to reflect a review of the victimization and help-seeking literature, given the large hole in the literature on help-seeking behaviors after community violence, we incorporated this inductive approach because we expected to identify categories, sub-concepts, and themes that did not fit within the extant models reviewed. Hence, we searched for gaps and worked together to clarify an understanding of new categories and sub-concepts where necessary. This iterative process allowed the research team to reach a consensus on the codebook similar to the cycle coding process set out by Saldana (2013). Inter-coder agreement also was built into the coding strategy, and segments were scored for agreement using a template similar to that suggested by MacQueen et al. (2008). Segments needing recoding were re-coded by both parties and re-scored until coders reached a threshold of 85% agreement.

## Findings

**Fig. 1:**
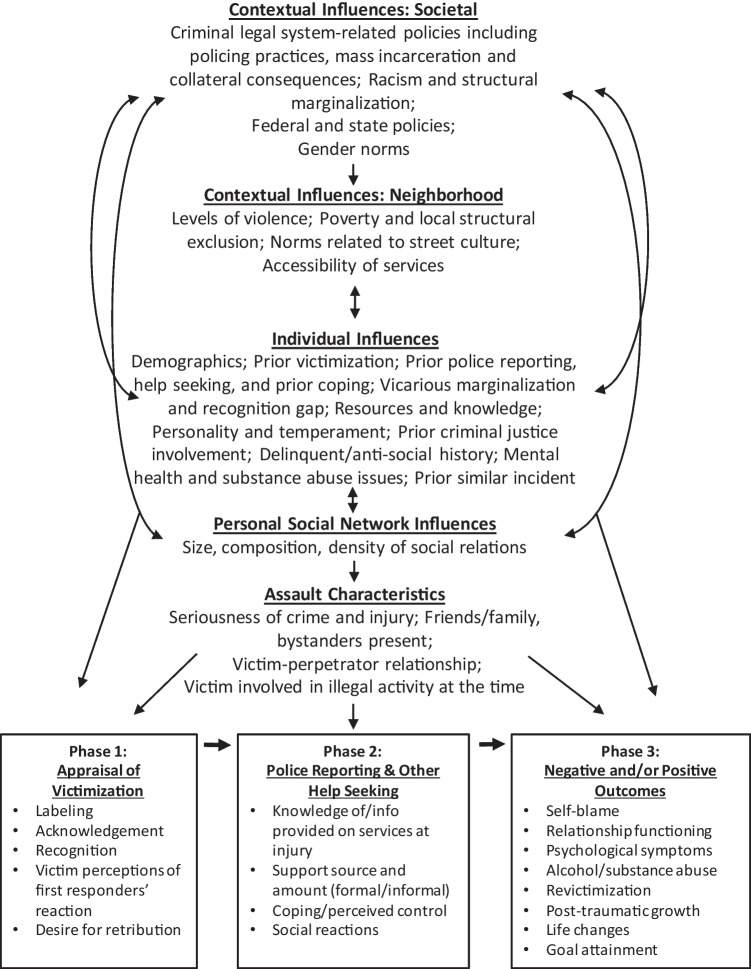
Adaptation of Ullman’s (2010) model of sexual assault disclosure and reporting to help-seeking by Black Americans injured by community violence

Data analysis to assess the frequency of codes and subcodes overwhelmingly supported the draft conceptual model that Black males’ formal help-seeking behaviors may be associated with a wide range of structural, neighborhood, individual, and assault-related factors. Below, I discuss the findings regarding the salient pre-victimization and assault-related factors and, where relevant, the main themes and subthemes that emerged within the related codes and subcodes, with specific attention to aspects of help-seeking that did not align with the draft model. Table 1 shows the frequency of code/subcode application for the pre-victimization and assault levels of influence and associated factors, as well at the percentage of respondents represented. These codes and code groupings in Table 1 represent the structure of the coding framework model (i.e., the draft model), not the final model. Figure 1 details the final comprehensive conceptual model after analyses of data and consideration of findings. The Figure also depicts the three phases of post-victimization help-seeking not discussed in this article.

**Table 1:** Code structure, description, and code frequencies for the draft help-seeking model

**Categories of influence and related aspect**	**Description**	**Code Freq**	***N*** **(%) of victims**
**Contextual: social structure**			
Criminal justice-related	Perception of nature of CJ agents, justice system, mass incarceration, etc., affects life; myths re: victim blaming were also coded here	44	26 (31.9%)
Economics	Perception of how systemic/macro-economic constraints affects life in general	34	5 (5.5)
Resources	Perception of how (lack of) resources affects life in general	12	10 (11.0)
Federal & state policies	Perception that laws and policies play a role in life outcomes	0	0 (0)
**Contextual: culture**			
Racism/race & ethnicity	Cultural influences related to race or ethnicity; includes overt mention of racism or race when discussing response/interaction with police or other formal institutions	8	5 (5.5)
Neighborhood	Patterns or characteristics of human activity that reflect the neighborhood as a group. Can be positive or negative	79	45 (49.5)
Gender norms	Gender-related beliefs which shapes R’s behavior or the victimization incident	9	5(5.5)
**Individual**			
Race/ethnicity	Comments related to R’s own race/ethnicity that may be linked to help-seeking	6	6 (6.6)
Gender	Comments related to R’s gender	2	1 (1.1)
Employment/occupation	Comments related to R’s job or work status (including unemployment)	5	4 (4.4)
SES	Comments related to R’s socioeconomic status	2	1 (1.1)
Marital status	Comments related to R’s marital status	0	0 (0)
Geographical	Comments related to where R lives (different from neighborhood cultural aspects)	3	3 (3.3)
Prior victimization: self	Comments about personally experiencing prior victimization	25	16 (17.6)
Prior victimization: other	Comments about witnessing victimization of others	4	3 (3.3)
Prior victimization disclosure	Comments about previously experiencing disclosure of victimization to any potential support source, informal or formal	20	10 (11.0)
Individual resources	Financial, human, etc., resources/knowledge related to accessing services. Includes interpersonal networks	37	22 (24.2)
Personality trait	References to R’s own personality or temperament	26	22 (24.2)
Delinq./anti-social history	References to past delinquent/anti-social behavior (not involvement with CJ system)	6	5(5.5)
Mental health/substance abuse issues	References to past issues with mental health or substance abuse that affected behavior	8	7 (7.7)
Prior criminal legal system involvement^a^	Specific mention of past involvement with the criminal legal system	82	42 (46.2)
Prior similar incident	Vague references to involvement in anti-social or negative incidents in the past	18	14 (15.4)
Prior coping process: adaptive	References to positive means of coping (e.g., seeking social support, reducing stressors)	2	2 (2.2)
Prior coping process: maladaptive	References to negative means of coping are “maladaptive” behaviors such as withdrawal, denial, disengagement, substance use, dangerous behaviors	12	9 (9.9)
**Assault characteristics**			
Seriousness of injury	Specific references to seriousness of the injury (not the criminal offense)	96	79.12%
Victim-perp.relationship	References to the relationship between R and perpetrator	89	75.82%
Friends/family at scene	Mention that family or friends were at the scene of the victimization	86	57.14%
Precipitating events	References to events that led to victimization (important to understanding injury)	72	72.53%
Bystanders at scene	Mention that bystanders were at the scene of the victimization	41	32.97%
Crime seriousness	Comments about seriousness of what happened/weapons involved	38	35.16%
Victim involved	Any mention that R was involved in some kind of illegal activity at the time	18	16.48%

*R* respondent

^a^Prior criminal legal involvement includes any contact with the criminal legal system, including comments that the respondent had been arrested in the past, been in prison is, or was on probation or parole, etc

Contextual influences are the social, political, physical, and economic predictors encompassing the broader societal structural and local neighborhood context that interact with individual-level characteristics to shape behavior related to reporting victimization and seeking help. The draft model/codebook had originally separated contextual influences into two areas: social structural versus cultural, but many excerpts were often coded as reflecting both areas and the distinction did not neatly capture some of the nuances of local neighborhood attributes and culture. The final conceptual model (Fig. 1) collapsed the larger macro-level forces of systemic racism and marginalization, criminal justice policies (and other policies), and societal norms into one level of influence ("societal"), separating them from the more-local factors associated with neighborhoods. The array of societal and neighborhood contextual factors pervade the whole help-seeking process (i.e., all phases) and interact with each other.

### Contextual Influences

As shown in Table 1, roughly one-third of respondents mentioned societal-level contextual “criminal justice-related” factors related to mass incarceration, criminal legal system processing, and general inequities in police responses; these issues were the most salient aspect of respondents’ comments with regard to responses to their victimization and their subsequent paths to care. Respondents specifically mentioned inequalities in the legal system playing a role in increasing their distrust in criminal legal system agencies and actors, which then influenced their immediate appraisal of their victimization (help-seeking phase 1) and whether they talked to the police or sought criminal justice services (help-seeking phase 2). These appraisals and decisions often depended on the specifics of the assault. In addition, some respondents discussed not trusting the police, but also recognized that their attitude and behavior at the scene might negatively influence whether the police would assist them, follow-up on the incident, and help arrest the perpetrator. One respondent’s comments sums this up: “Where I’m from, the police ain’t our friend. Cut and drop, they’re not our friend so we don't go to them for nothing…For anything. They don’t do nothing. When they come take the report, they give you the impression or the feeling that he’s just writing it up.” A number of respondents mentioned that police responders immediately engaged in victim blaming or likely did not offer information on victim services because they believed the victim was partially to blame. One respondent when asked why he thought he wasn’t given information on victim services, responded:

Respondent: Probably because I’ve been shot four different occasions.

Interviewer: They saw your record…?

Respondent: Yeah, [they were thinking] he’s not going to learn, yeah…

Another respondent, a 33-year-old male stabbing victim, explained the police practice of “dry snitching” and stated he would not get involved with police or victim services because it would put him at risk of the police implicating him as a snitch even if he didn’t provide any information to the police. Dry snitching is a slang term used here to describe police implicating someone as a snitch without directly using their name.

The next most-discussed contextual aspect across respondents was the general low level of economic resources in society and Philadelphia overall (10% of respondents), with some specifically mentioning the systemic nature of disinvestment in communities or referred to social stratification (5%), a topic that aligns with aspects of Bell’s legal estrangement theory. One young mother who was assaulted in her front yard, in describing her experiences with the responding police officers (who did not provide her with information on victim services) said:

“I felt l﻿ike they weren’t trying to hear my story. They already had got the story from her [the perpetrator] and that’s always like when I’m going through something legal. The perp will get to them first and make me look bad…I just withdraw. People with financial means and everything they always going to win. Kick around the little guy. That’s how I do feel with some law enforcement… they made me feel like I’m just some ghetto loudmouth chick. They was, ‘We don’t even want to hear it.’”

Five respondents specifically named racism by the police as an issue. One 18-year-old female who was beaten up at school went to the police district headquarters with her mom the next day with the intent to press charges but reported in her interview that the police were dismissive and withheld information on how to press charges. She attributed the dismissive attitudes as due to her being Black. She felt despondent and confused at the reactions by law enforcement and did not end up pressing charges.

In the context of respondents describing the police interaction and how that may have influenced victim decision-making and help-seeking, neighborhood characteristics tended to dominate the interviews, with the “neighborhood” code applied 79 times across 45 respondents. A close examination of excerpts revealed the following themes: (1) high-crime neighborhoods draw the attention of the police, (2) perceptions of local structural exclusion and resulting recognition gaps (individual level), and (3) presence of a street norms/culture thwarts any benefits that might be gained by police reporting or seeking help from victim services. Many of these themes overlapped with the larger societal themes of racism and criminal legal system injustices.

Respondents were pointed in their comments that attributes of their neighborhoods brought the attention of law enforcement. Some of these comments either directly or indirectly referred to race and/or racism, where, as Bell (2020) articulates, individuals recognize that legal authorities are treating them (as a group) different. Bell refers to this perception—where individuals recognize that a group with power is treating less powerful groups inequitably—as “recognition gaps.” These recognition gaps are individual-level influences (i.e., perceptions held by the individual) but also tap into reflections about one’s neighborhood. Often these comments respondents made about legal authorities reacting inequitably also are entwined with the larger macro-economic/societal context. One 23-year-old male stabbing victim stated:

See a lot of times, like one thing I realize, I live in, you know, a pretty poverty-stricken neighborhood technically speaking… I don’t live in the projects, I live right there [points] which doesn’t really make a difference… so more than twenty feet away I’d look like anybody you know my status, skin complexion everything so I get harassed by undercover cops, I get harassed by regular cops… I got stopped by a [university] cop before when I was in high school because I actually went to [high school nearby] and somebody had just robbed a [university] student and then the cops like they, they stopped me and was looking at me and it was just like I got my gym shirt on, I didn’t rob anybody.

Importantly, discussions that touched on recognition gaps also evoked tit-for-tat type beliefs and comments where respondents often stated that if police were not going to try to catch the perpetrator, the respondent was not going to cooperate with the police. Some respondents knew about the possibility of receiving free victim services and compensation but also knew that meant a police report had to be generated and bluntly stated during interviews that they wouldn’t get involved. One respondent’s remark summed up this theme: “It’s streets. Whatever happens in the streets stays in the streets.” This street code essentially curtailed respondents’ interest of engaging with the police and victim services. With the exception of one victim whose mother was a local police officer, no respondents mentioned their neighborhoods as places with strong local networks that enabled positive engagement with government agencies.

A few male respondents (5.5%) discussed being able to handle the pain of the injury, and even though the pain was bad, they didn’t need medical treatment. These comments were coded as aspects of gender dynamics related to toughness or masculinity. These respondents indicated that they were “fine” after their serious injury and stated that their injury must be viewed as severe to justify needing help. Pain was normalized. Even when male victims called the police, they appeared conflicted about it, and their narratives suggest they wanted to portray themselves as strong, “deal with the problem” themselves, but believed they needed to do the right thing by reporting the crime. It is also possible that the passages we coded as gender norms could be coded as norms around Black male toughness and masculinity (St. Vil, 2020), or simply could represent respondents’ unwillingness to report other concerns such as cost, or concerns about fair treatment. Although the draft model included the aspect of *federal and state policies*, no respondent specifically mentioned any official state or local law or policy (note, the policy that states uncooperative victims can be barred from victim services was not included under this code but coded as *criminal justice-related*). This factor will remain part of the conceptual model, given it was found to be relevant in the literature review.

### Individual-Level Influences

At the individual level, the draft model included age, race, gender/gender identity, employment/occupation, SES, marital status, geography, and individual-level access to and knowledge of services and resources, prior victimization, and prior disclosure and help-seeking. Marital status and specific geography/neighborhood location did not emerge as important factors from the analyses—only three individuals mentioned geography, and no respondent referred to their marital status. Being Black was only mentioned explicitly by six victims during the interviews, and most of these excerpts were part of larger excerpts detailing perceived acts of racist treatment or discrimination (also coded under cultural context).

The most frequently used codes and subcodes related to individual influences were, in order of frequency as follows: (1) prior criminal legal system involvement (82 excerpts/46.2% of respondents); (2) individual knowledge and resources, including support from close social network (37 excerpts/ 24.2% of respondents); (3) personality traits and/or temperament (26 excerpts/24.2% of respondents); (4) prior victimization-self (25 excerpts/17.6% of respondents); (5) similar type of incident (18 excerpts/15.4% of respondents); and (6) prior maladaptive coping process (18 excerpts/9.9% of respondents). Within “knowledge and resources,” having supportive family members appeared to reduce the likelihood a respondent would turn to formal institutions for help. Notably, there were numerous mentions by respondents of having specific personality traits or temperament (the third most frequent individual-level factor, as listed above) that influenced help-seeking behavior. Interestingly, a number of these excerpts had to do with respondents describing themselves as detached and having a desire to avoid chaos. One 29-year-old Black female who had been in a bad fight, when asked why she would never call the police, replied: “Because I’m not the type of person to be out here going back and forth to court… I don’t want to be back and forth to court and then I go to court and probably she’s going to have family be coming at my family then there’d be a whole lot of chaos and I don’t got time for it so I just let it be.” For some respondents, comments were related to prior *adaptive* coping processes, indicating they drew on their knowledge that they knew how to positively cope with adverse events, including any continuing harm or sequelae related to the injury.

Other important individual-level factors that arose from the analyses included having witnessed the victimization or the effects of victimization of loved ones or hearing about the experiences of police maltreatment or unequal treatment by any government agency toward family and friends in the respondent’s personal network. The latter—hearing about unequal treatment of Black men and women by government agencies, dovetails with Bell’s (2017) concept of “vicarious marginalization.”

An analysis of excerpts coded for any mention of a victim having a history with the criminal legal system yielded a main finding that victims who had any previous contact with law enforcement involving law breaking by the victim were sure they would be treated unfairly or blamed for the incident if they were to contact the police. This code often overlapped with both societal racism and neighborhood exclusion. One respondent said:

“Because for me, going to the hospital, calling the cops…I actually have to think about things before…especially am I going to be able to pay the [expletive] bill…when I was growing up, we didn’t have no money…my dad always used to tell me, don’t go to the emergency room, So initially you don’t even think of doing that when you first get hurt. You think of taking the pain…And especially police, in these days considering who I am and what I am, I’ve got to think twice because it’s not just immediately thinking that if I’m in a situation and I call the police they’re going to be on my side, especially considering I have a record.”

One male victim of assault who admitted he knew about victim services from watching the television show *Law & Order: Special Victims Unit* stated that although he knew he could be offered services, he wanted to leave the hospital as quickly as possible even if it meant not being offered services.

For those who indicated the police treated them unfairly or without respect in the past, there appeared to be a cumulative effect. Repeated instances of injustices by the police greatly dissuaded any current legal involvement—from calling 911 to interacting with police if they arrived on the scene or came to the hospital. And nine victims (10.0%) specifically discussed poor treatment by police responders during their current interaction with the police and attributed that poor treatment to bias against them because of their previous criminal record or because they were still under supervision. Respondents who were currently on probation or parole reported particularly unfriendly interactions with the police related to the current victimization, which tended, in turn, to make them firmly opposed to sharing information to the police about the incident and (in the cases where hospitals had victim advocates) reluctant to accept information hospital-based victim advocates may have offered. A few of these individuals indicated that they had intended to cooperate, but the negative interaction changed their minds. When the research interviewer asked respondents if they would have cooperated had they known about victim services (and the requirement of cooperation), most said they might have changed their minds, but they still did not quite believe that police were supposed to (and would) support them. These beliefs held by victims that they would not be able to access victim services even if they cooperated were espoused by a number of respondents. One 25-year-old shooting victim with an arrest history, who discussed avoiding formal support systems, including victim services, said police would likely accuse him of something else if he sought out help:

It’s just basically like cause it was just because of my history like they probably just knew of me. Like, that’s not my neighborhood that I’m from, but since I got locked up and running now… they probably feel as though they know me… I got locked up around there, I [must be] from the neighborhood. If it’s a little gang around there, they going to put me in there just because I live around there and I got locked up. So, they going to accuse me as a gang member.

The quote above also shows that one’s personal history of criminal behavior is intertwined with officers’ racial and cultural biases about certain neighborhoods. This interaction aligns with Ford’s (1995) concept of “racially identified spaces,” with police holding a negative view of people living in certain neighborhoods. The point here is that the *assumptions* victims have about police reactions create a large barrier to victim services both directly and indirectly.

Prior victimization was also a highly relevant theme in our findings in that a few respondents indicated that law enforcement would arrive on the scene and recognize them as having a history of involvement in street assaults and instantly take on an unsupportive attitude toward them. Victims also indicated, however, that prior victimization may increase help-seeking in that victims have more opportunities (through repeated contact) to learn about available victim services, a finding that aligns with a past study (Fohring, 2015).

### Assault Characteristics

A wide array of characteristics of the victimization incident itself (“assault characteristics”) emerged as factors that appear to influence the victim’s decision to involve the police, cooperate, and/or seek victim support and related social services. The top five characteristics, in order of frequency, were (1) seriousness of injury, (2) relationship between victim and perpetrator, (3) if family and/or friends were present at the scene of the incident, (4) the events that precipitated the crime, and (5) bystanders were present. Each of these themes was associated with more than 30 excerpts, with the seriousness of the injury referenced 96 times. Some respondents discussed, for many reasons, that they wanted to avoid interaction with the police and other formal institutions, such as the hospital, if the assault was related to a gang feud, ongoing neighborhood beef, or drug-sales, even if the respondent was not involved in illegal activity at the time (such as the 33-year-old respondent who discussed the police and dry snitching). The large number of factors present under this category led to modifying the original “levels of influence” to draw out “assault-level” characteristics to stand as its own level of influence, apart from the societal-, neighborhood-, and individual-level categories (see Fig. 1).

## Discussion

Overall, similar to drawing out assault-level factors as its own category, the frequency in which respondents discussed themes related to their neighborhood as relevant to their help-seeking paths suggests that the final conceptual model should be modified (from the coding framework model) to accommodate these findings. In addition, given that there were a large number of excerpts related to *interpersonal* resources (i.e., informal supports) within the code “individual: resources,” the analysis of themes suggests that one’s social network might be better served in a framework as it is own level of influence (refer to Fig. 1). The particular themes uncovered under this umbrella included the size of one’s social network, composition, and cohesion of one’s network, particularly family—not needing to engage with formal institutions because of a cohesive family network of supports. Furthermore, analyses of the data as described above highlight the importance of vicarious victimization and vicarious marginalization—processes dependent on one’s social network (and the composition of the network) and perhaps may be more appropriately studied at the relationship/social network level. Certainly, the use of social network analytical methods to understand how networks and relationships influence health outcomes is growing rapidly; thus, calling out social networks as a separate component might help a facilitate a nuanced examination of the influence of informal networks.

The final conceptual model of Black victim help-seeking has five pre-victimization levels of influence each with their own factors, as well as three phases of help-seeking (the latter not addressed in this article). The model is heuristic; it does not identify specific pathways or interactions. The arrows are not meant to be definitive but merely suggestive of possible directions of relationships. The model was developed to encourage research and practice that can systematically assess, and perhaps disentangle, the many influences on the process, structure, and outcomes related to accessing victim services and care for Black Americans who have been injured by violence. Distinction by levels of influence will facilitate nuanced and valid multi-level measurement and appropriate research methodologies applied to examine the factors and, hence, have implications for prevention and intervention that are relevant to each level. In addition, attention to the conceptual model hopefully will promote access to and the use of data and measures that systematically account for cultural context at both the societal and neighborhood levels. Research that undercovers causal linkages among macro-level factors and Black American health outcomes can promote institutional, community, and government policies that move the needle from intervention to prevention and help further physical, social, and emotional well-being. The separation of levels of influence also will provide some insight on the likelihood that prevention levers could be scaled, a key aspect of prevention science.

The findings from the analyses of respondent narratives highlight that there are a wide array of societal factors that likely interact with individual characteristics to limit help-seeking behaviors, but on a positive note, the findings also reveal mutable factors that can be addressed to prevent short- and long-term harms. For instance, at the societal-structural level, efforts to convene councils or workgroups comprised practitioners and policy leaders, where physicians and trauma surgeons present and review cases that highlight how structural barriers may lead to violence and trauma and can help raise awareness and empathy and reduce implicit and explicit biases that disrupt trust. Also at a societal level, public educational campaigns explaining victim rights and victim compensation programs may increase access to services. Focused police policies supporting police advocacy for victims’ rights, such as the use of checklists in police procedure during interactions with victims and repeated specialized training on victims’ rights and implicit biases can improve police–victim interactions. Given that a large number of respondents’ perceived deep biases and racism by first responders and police detectives, policies and programs that support true community policing ideals of building community trust and legitimacy through regular community outreach and dialogue and transparency and accounting of policing activities may reduce legal estrangement, perhaps with long-term implications for minimizing the cycle of violence and retaliation.

Targeted approaches through general victim outreach, or hospital violence intervention paired with community health workers who have lived experiences of violence, can support not only awareness of and access to and longer-term engagement in services, but also building trust and positive messaging about beneficial services. Along this line, Alvidrez et al. (2008), evaluating a victim assistance program, found that augmenting general knowledge of victim services increased the proportion of victims filing financial compensation claims and also reduced disparities. Furthermore, the findings that many respondents believed their neighborhoods were over-policed and under-resourced imply that increasing funding for and the visibility of neighborhood-based victim services may be restorative for residents.

There are limitations that need to be mentioned. First, the findings only reflect the perceptions of the survivors of violence—this is only one side of a multi-sided interaction that includes police first responders, victim services, and hospital personnel. Second, these findings are based on a purposive sample of Black men and women injured in violent incidents living in a high-crime city, so the findings cannot be generalized or compared against perceptions of non-Black victims, or those residing in suburban or rural areas. Future research should seek these comparisons. Third, the conceptual model does not incorporate aspects of ethnicity and shared heritage to examine intra-group differences. In essence, more work is needed to test the robustness of the model.

## Conclusion

Help-seeking is a multi-faceted and complex behavior. The attainment of effective formal help that meets the needs of Black Americans is likely to increase positive health outcomes and reduce health disparities. The current gap in the victimization literature is startling given the toll violence exacts on Black communities. Also surprising is that some of the potential policy levers to reduce racial disparities in service access may be relatively simple or inexpensive to fix. Although intervening at the societal level is most difficult, the findings suggest there is great potential for some barriers to be overcome, for instance, through the highly mutable factor of individual knowledge and awareness. In short, the path to preventing some future harms from victimization in Black communities seems within our reach.
